# Supplementary material for: A high-resolution mRNA expression time course of embryonic development in zebrafish
Source: eLife. 2017 Nov 16;6:e30860. doi: 10.7554/eLife.30860 (PMC5690287; doi:10.7554/eLife.30860)
Supplement: Supplementary file 6. [file elife-30860-supp6.zip › biolayout-clusters-files/Cluster110-genes.html]

Cluster110


# Cluster110: Genes

| Ensembl ID | Gene Name | Chr | Start | End | Biotype |
| --- | --- | --- | --- | --- | --- |
| ENSDARG00000075274 | ENSDARG00000075274 | 25 | 2340579 | 2358278 | protein\_coding |
| ENSDARG00000061131 | kif21a | 25 | 35179017 | 35260985 | protein\_coding |
| ENSDARG00000039066 | klhl31 | 13 | 2303263 | 2315264 | protein\_coding |
| ENSDARG00000102100 | mrc1a | 7 | 58589600 | 58650000 | protein\_coding |
| ENSDARG00000100398 | pax7a | 11 | 40652295 | 40787221 | protein\_coding |
| ENSDARG00000000369 | prkag3b | 9 | 14119126 | 14137693 | protein\_coding |
| ENSDARG00000088881 | si:dkeyp-69b9.3 | 16 | 13614899 | 13652395 | protein\_coding |
| ENSDARG00000099959 | smyhc1 | 24 | 40956288 | 40968424 | protein\_coding |
| ENSDARG00000005916 | srpk3 | 8 | 8252907 | 8300787 | protein\_coding |
| ENSDARG00000076241 | txlnbb | 17 | 6200930 | 6225162 | protein\_coding |
| ENSDARG00000079198 | usp13 | 6 | 29420425 | 29478647 | protein\_coding |
| ENSDARG00000010008 | vim | 24 | 32261512 | 32278038 | protein\_coding |
